# Recurrent Coagulopathy after Rattlesnake Bite Requiring Continuous Intravenous Dosing of Antivenom

**DOI:** 10.1155/2015/719302

**Published:** 2015-01-12

**Authors:** Charles W. Hwang, F. Eike Flach

**Affiliations:** Department of Emergency Medicine, University of Florida College of Medicine, 1329 SW 16th Street, P.O. Box 100186, Gainesville, FL 32610-0186, USA

## Abstract

*Context*. Snakebite envenomation is common and may result in systemic coagulopathy. Antivenom can correct resulting laboratory abnormalities; however, despite antivenom use, coagulopathy may recur, persist, or result in death after a latency period. *Case Details*. A 50-year-old previously healthy man presented to the emergency department after a rattlesnake bite to his right upper extremity. His presentation was complicated by significant glossal and oropharyngeal edema requiring emergent cricothyrotomy. His clinical course rapidly improved with the administration of snake antivenom (FabAV); the oropharyngeal and upper extremity edema resolved within several days. However, over the subsequent two weeks, he continued to have refractory coagulopathy requiring multiple units of antivenom. The coagulopathy finally resolved after starting a continuous antivenom infusion. *Discussion*. Envenomation may result in latent venom release from soft tissue depots that can last for two weeks. This case report illustrates the importance of close hemodynamic and laboratory monitoring after snakebites and describes the administration of continuous antivenom infusion, instead of multidose bolus, to neutralize latent venom release and correct residual coagulopathy.

## 1. Introduction

The Crotalinae subfamily of snakes (family Viperidae), commonly known as pit viper snakes, include rattlesnakes (*Crotalus* species), pygmy rattlesnakes (*Sistrurus* species), and moccasins (*Agkistrodon* species) [1]. Pit viper envenomations are not uncommon in the United States. Annually, approximately 2,700 envenomations in the United States lead to hospital presentation; half of them receive the antidote for Crotalinae envenomation, Crotalidae Polyvalent Immune Fab (Ovine) (FabAV) (CroFab, Protherics, Nashville, TN) [1, 2].

Snakebite envenomation causes not only localized tissue damage, but also systemic derangements. One of the well-known sequelae after snakebites is the systemic coagulopathy due to enzymes within the venom, which result in laboratory abnormalities, including elevated d-dimer, hypofibrinogenemia, prolonged prothrombin time (PT), prolonged activated partial thromboplastin time (aPTT), and thrombocytopenia. The use of antivenom can correct these laboratory abnormalities; however variable response has also been observed; the coagulopathy may recur, persist, or result in death after a latency period [3, 4]. Therefore, the administration of antivenom must be tailored to each patient's clinical and laboratory presentation and venom exposure [1].

The manufacturer and the local poison center recommend initial boluses of CroFab with subsequent maintenance boluses as needed until initial control is achieved. In this case report, we describe a patient with recurrent coagulopathy after a snakebite that ultimately required 51 vials of Crofab. Furthermore, the coagulopathy resolved only after a continuous intravenous infusion of CroFab was administered.

## 2. Case Presentation

**Figure 1:**
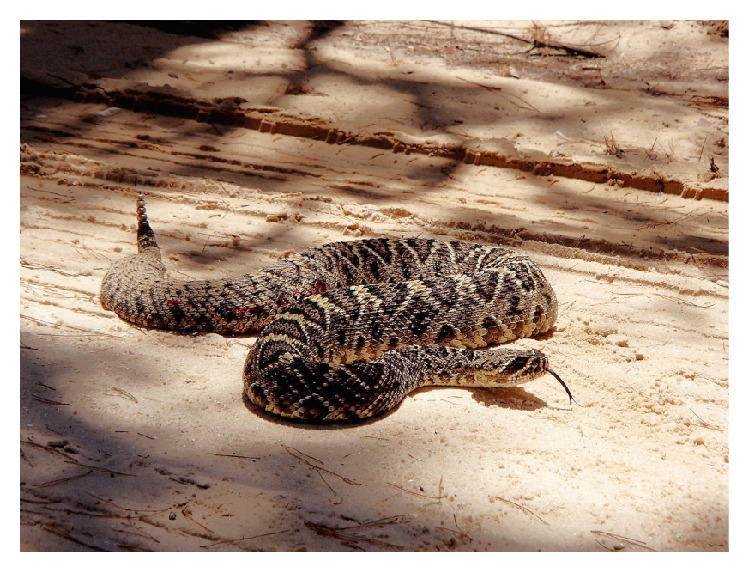
Photograph of the eastern diamondback rattlesnake (*Crotalus adamanteus*) taken by the patient's wife after the patient's snakebite.

A previously healthy 50-year-old intoxicated man was driving on a road on a May afternoon when he attempted to move a snake off the road with a stick to prevent it from being run over. He was bitten on the dorsum of his right hand by a positively identified 6-foot long diamondback rattlesnake (Figure 1). He immediately called his wife who called emergency medical services (EMS) while he drove home. When EMS arrived, he was found to be confused, nauseated, and vomiting, with altered mental status and intermittent combativeness. Once in the transport vehicle, an intravenous line was established and he was given 25 mg of intravenous (IV) diphenhydramine.

Upon arrival in the emergency department (ED) less than one hour after the initial injury, the patient was noted to be tachycardic (HR 131), hypotensive (92/79), and stridorous. On physical examination, he had decreased mental status, voice change, and significant perioral, pharyngeal, and marked glossal edema. Rapid sequence intubation (RSI) was initiated due to impending complete upper airway obstruction. The patient was pretreated with 100 mcg of phenylephrine IV to prevent hemodynamic decompensation during RSI. During video-assisted laryngoscopy, pronounced epiglottal and cord edema was noticed, and multiple attempts of passing a styletted endotracheal tube and gum elastic bougie failed. Bag valve mask ventilation was difficult despite use of an oral airway with a decline in his oxygen saturation to 78%. Thus the decision was made to proceed with emergency cricothyrotomy using a bougie-assisted landmark-guided technique, which was successful on its first attempt. A cuffed 6-0 endotracheal tube was passed over the bougie, with good chest rise and end-tidal capnography. At the time of securing the airway, the patient's oxygen saturation had returned to 100% secondary to continued uninterrupted two-person oral airway assisted bag valve mask ventilation.

Initial arterial blood gas analysis showed a pH of 7.03, pCO_2_ of 56.1 mmHg, and pO_2_ of 174.0 mmHg on 60% FiO_2_. He received 125 mg IV methylprednisolone and 1 L Plasma-Lyte A with improvement of his blood pressure to 119/76.

**Figure 2:**
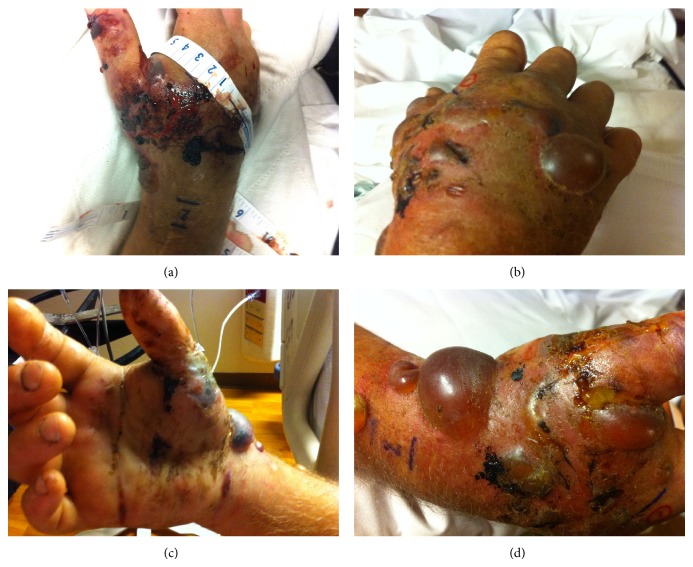
Fang marks located on the patient's right upper extremity with local tissue damage, edema, ecchymoses, and mottling.

His right upper extremity was noted to have fang marks 2.5 cm apart in the first dorsal webspace (Figure 2). There was severe amount of edema over the palmar and dorsal surface of the right hand extending proximally to the wrist with mottling and ecchymoses of the right hand. The compartments remained compressible, capillary refill remained brisk, and oxygen saturation remained 95–99% SpO_2_ in all five fingers. Orthopedic surgery was consulted by the emergency physicians for the evaluation of progressive swelling and potential compartment syndrome; no surgical intervention was performed as the patient maintained a radial pulse by Doppler signal and brisk capillary refill.

The state's poison center was simultaneously consulted in the ED; an initial bolus of 6 vials of FabAV (CroFab) was administered. Initial pre-FabAV laboratory findings from the emergency department demonstrated consumptive coagulopathy: thrombocytopenia (platelets 20 × 10^3^ mm^−3^), d-dimer > 20 *μ*g mL^−1^, fibrinogen < 35 mg dL^−1^, INR > 8, PTT > 240 sec, and PT > 150 sec. The patient was subsequently admitted to the medical intensive care unit for further management.

The state's poison control recommended boluses of CroFab per their protocol, which initially corrected his coagulopathy. His coagulation panel at the time of administration of FabAV throughout his hospitalization is shown in Table 1. The first week of his hospitalization was uneventful. The edema of his right upper extremity stabilized within 24 hours and did not require surgical intervention. He was successfully extubated on day 5.

On day 8 of his hospitalization, his fibrinogen and platelet count trended downwards, and his PT and INR trended upwards. He remained hemodynamically stable with no drop in his blood pressure, hemoglobin, or hematocrit and did not exhibit signs or symptoms of bleeding from his coagulopathy. There was no recurrence of swelling. Hematology was consulted, and decision was made to start him on FabAV infusions each over 6 hours instead of 1 hour. After six vials of FabAV were infused over 6 hours each, his coagulopathy resolved. The resolution of his coagulopathy is demonstrated in Table 1. His coagulopathy resolved by day 12 and he was subsequently discharged from the hospital.

## 3. Discussion

Snakebite envenomation is not an uncommon occurrence in the United States. In the United States, 8,000 poisonous snakebites occur annually, which result in 9 to 15 fatalities [5]. Envenomation causes localized tissue damage, which may manifest as fang puncture, pain, tissue edema, erythema, ecchymosis, bullae formation, and lymphadenopathy. In addition, systemic effects after envenomation include panic and fear, nausea, vomiting, diarrhea, lymphadenopathy, syncope, tachycardia, hemorrhage, hypotension, tachypnea, respiratory distress and failure, coagulopathy, and encephalopathy [4–7].

The toxic effects of venom assist in its function to obtain food for the snake. The enzymes it contains help to decrease digestive time and to immobilize the snake's prey. These enzymes alter the endothelial lining, break down plasma membranes, and promote edema and hemorrhage. Therefore, when humans are subjected to snake venom, hypovolemic shock, pulmonary edema, tissue necrosis, and renal failure ensue [5].

For many years, the coagulopathy after snakebites has been observed in vivo and in vitro, resulting in hemorrhagic and thrombotic events, with or without laboratory perturbations, due to activation of specific anticoagulant and/or procoagulant pathways [3, 8]. Thrombin-like and proteolytic enzymes contained within snake venom incompletely split the fibrinogen molecule, resulting in an unstable fibrin clot which traps platelets. Plasmin lyses these clots, resulting in a disseminated intravascular coagulopathy- (DIC-) like picture, which includes prolonged clotting times, prolonged prothrombin and activated partial thromboplastin times, hypofibrinogenemia, thrombocytopenia, and fibrin degradation products [5]. The clinical importance of coagulopathy is incompletely understood. Despite the significant, and occasionally extreme, perturbations in laboratory coagulation panels, these alterations do not always translate to hemorrhagic risk and hemorrhagic events [3, 8].

In the past, the duration of coagulopathy after snakebite has traditionally been considered short-lived and patients were routinely discharged after initial correction of coagulopathy [1]. However, recent literature has demonstrated that after adequate initial antivenom therapy, recurrence of coagulopathy may occur for up to 2 weeks [1, 3, 9]. In a retrospective study by Bogdan et al., 45% of snakebite patients had recurrent coagulopathy, including hypofibrinogenemia or thrombocytopenia [10]. Boyer et al. described 53% of FabAV treated envenomations that had recurrent, persistent, or late coagulopathy [1]. Hardy et al. reported recurrent thrombocytopenia despite initial correction of coagulopathy [11]. Other authors have reported persistent thrombocytopenia despite antivenom treatment [12–14]. Even though such a large percentage of patients demonstrated persistent or recurrent coagulopathy in these studies, none had clinically significant bleeding from the coagulopathy, nor did they have progression of local injury.

The mechanism of recurrence is unclear. The half-life of FabAV is less than 12 hours. It has been hypothesized that depots of unneutralized venom may continue to be released into the circulation after antivenom levels fall causing recurrent coagulopathy. Another hypothesized mechanism is the dissociation of antivenom-venom complexes, similar to digoxin-specific Fab dissociation, causing a recrudescence of coagulopathy [1, 3, 15].

Again, the clinical significance of recurrent late coagulopathy is unclear. Some experts believe that because the coagulopathy is a result of defibrination syndrome, patients are not at increased risk of bleeding [2]. However, other experts hypothesize that patients are one step away from a catastrophic hemorrhage [1, 8]. Kitchens and Eskin reported a case of delayed, recurrent coagulopathy that resulted in a fatality due to devastating intracerebral hemorrhage [3]. Since (1) pharmacokinetics strongly argues in favor of maintenance therapy to prevent recurrent coagulopathy, (2) the clinical significance of coagulopathy resulting in hemorrhage is unknown, and (3) a catastrophic event could cause life-threatening hemorrhage, low fibrinogen levels, and prolonged clotting times, and thrombocytopenia should be considered potentially clinically significant, and recurrence should be managed with additional antivenom [1, 2].

In this case, our patient initially received boluses of FabAV per current prescribing guidelines. His local injury was well-controlled with no progression of swelling or extension after the first 24 hours. Despite initial correction of his coagulopathy, our patient developed recurrence of his coagulopathy on day 8. He did not demonstrate any local or systemic signs or symptoms of venom toxicity, nor did he have any clinically significant hemorrhage or hemodynamic instability secondary to coagulopathy; he remained hemodynamically stable with an intact airway and no worsening of his extremity edema despite his coagulopathy. Lavonas et al. and White hypothesized that antivenom redosing and maintenance dosing may be required in order to (1) provide sufficient antivenom to neutralize initial acute venom levels and to (2) neutralize latent venom release from soft tissue depots that can last for two weeks [2, 8]. The hematology service initially recommended an infusion regimen over 12 hours; however, because of the off-label administration regimen, the medicine, hematology, and pharmacy services jointly decided to instead administer the FabAV antivenom over six hours which rectified his coagulopathy. Within one day of initiating continuous FabAV infusion, the patient's hematologic derangements improved.

Bush et al. reported a retrospective case series of five patients envenomated by rattlesnakes with similar success. Despite initial bolus dosing of FabAV, the patients experienced either transient or inadequate response with profound delayed hematologic abnormalities. After initiating a continuous FabAV infusion at 2 to 4 vials per 24 hours, the hematologic derangements improved within six to fourteen days after initial injury [16].

In summary, snakebites cause in vivo and in vitro coagulopathy, which, at this point, has uncertain clinical significance with respect to hemorrhage. This coagulopathy can persist or recur up to two weeks after injury. Therefore, despite the unknown incidence of clinically significant bleeding, patients appear to be one step away from a catastrophic hemorrhage. At this time, there are many unknowns: the bleeding risk of delayed or recurrent snakebite coagulopathy, the consequences of prolonged antivenom administration, and the optimal rate of infusion to correct coagulopathy and prevent hypothetical thromboembolic events. It is uncertain whether any downsides exist for administering FabAV using maintenance dosing; we feel it would be prudent to monitor for thromboembolic events in the setting of coagulopathy. More importantly, we demonstrate in this case that maintenance dosing in the form of an infusion is a plausible modality of administration that may be considered in the management of serious Crotalinae envenomation complicated by coagulopathy.

## Figures and Tables

**Table 1:** Serial coagulation panel.

Day	1		2			3		4				5			6	
Time	1600	2259	0557	1415	2037	0411	1620	0004	0800	1535	2150	0353	1300	2359	0607	1210
Platelets × 10^3^ mm^−3^	20	493	528	416	352	293			200	263	335	341	219	141	115	116
PT, sec	150	29.8	16.9	15.5	15.6	15	14.6	15	15.5	15.1	15	14.6	14.3	15.7	15	14.5
INR	8	2.9	1.4	1.5	1.2	1.5	1.1	1.2	1.2	1.2	1.2	1.1	1.1	1.2	1.2	1.1
Fibrinogen, mg dL^−1^	35	35	104	167	176	166	158	164	151	129	156	150	189	232	291	319
D-dimer, *µ*g mL^−1^	20	20	20				16.93	12.58	17.27	20	20	17.05	12.38	8.8	10.16	10.9
CroFab, time, and vials	1700 12		0715 2	1845 2					1342 4	1939 4	0152 2	0658 2			1048 1	1628 1
	2036 6		1220 2													2229 1
	2246 6															

Day	7		8			9		10		11			12	19		

Time	0005	1215	0000	1145	2005	0410	1246	0405	1810	0535	1315	1715	0640	0907		
Platelets × 10^3^ mm^−3^	97	97	91	77	67	43	48	60	109	128	150		245	612		
PT, sec	15.4	15	15.4	15.2	17.1	17.4	15.9	15.9	15.5	15.4	15.3	14.6	14.4	13		
INR	1.2	1.2	1.2	1.2	1.4	1.4	1.3	1.3	1.2	1.2	1.2	1.1	1.1	1		
Fibrinogen, mg dL^−1^	394	452	495	305	110	111	183	195	314	243	261	267	282	464		
D-dimer, *µ*g mL^−1^	8.17										4.53					
CroFab, time, and vials					2241 1^†^	0520 1^†^	2331 1^†^	1100 1^†^	2235 1^†^							
						1048 1^†^										

^†^CroFab infusion over 6 hours.
